# Status of self-medication and the relevant factors regarding drug efficacy and safety as important considerations among adolescents aged 12–18 in China: a cross-sectional study

**DOI:** 10.1038/s41598-024-59204-2

**Published:** 2024-05-01

**Authors:** Diyue Liu, Pu Ge, Xialei Li, Wenying Hong, Mengjie Huang, Lijun Zhu, Ayidana Kaierdebieke, Wenbian Yu, Jiale Qi, Keping Pu, Rong Ling, LuTong Pan, Xinying Sun, Yibo Wu, Qiqin Feng

**Affiliations:** 1 International School of Public Health and One Health, Hainan Medical University, Hiaikou, China; 2 School of Traditional Chinese Medicine, Beijing University of Chinese Medicine, Beijing, China; 3 School of Pharmaceutical Sciences, Shandong University, Jinan, China; 4 University of Macau, Macao, China; 5 School of Public Health, ShanDong University, Jinan, China; 6 College of Communication and Art Design, University of Shanghai for Science and Technology, Shanghai, China; 7 School of Public Health, Xi’an Jiaotong University, Xi’an, China; 8 Zhuhai Institute of Social Development, Zhuhai, China; 9 International School of Journalism and Communication, Zhengzhou University, Zhengzhou, China; 10 Institute of School of Nursing, China Medical University, Shenyang, China; 11 Jilin University School of Pharmaceutical Sciences, Jilin University, Changchun, China; 12 School of Public Health, Shandong University, Beijing, China; 13 School of Public Health, Peking University, Beijing, China

**Keywords:** China, Adolescents, Self-medication, Efficacy, Safety, Health care, Public health

## Abstract

The objective of this study was to investigate self-medication behavior among Chinese adolescents aged 12–18 years and explore the factors associated with whether adolescents prioritize drug efficacy or safety when engaging in self-medication behavior. In 2021, a questionnaire investigation was conducted in the Chinese mainland using a multi-stage sampling approach. After a statistical description, logistic regression was used to analyze the factors associated with considering drug efficacy and safety. The self-medication rate among Chinese adolescents aged 12–18 years was 96.61%. Of these, 65.84% considered drug safety to be essential, while 58.72% prioritized drug efficacy. Regression analysis showed that individuals with better healthcare were more likely to consider drug efficacy an important factor. Additionally, those with a healthier family lifestyle were more likely to prioritize efficacy. When individuals engage in self-medication, those residing in urban areas and possessing advanced preventive health literacy and ample family health resources tend to prioritize drug safety to a greater extent. Conversely, those with higher monthly household incomes and only children exhibit a decreased inclination towards prioritizing safety during self-medication. Self-medication is a frequently observed practice among Chinese adolescents aged 12–18. Several factors, such as demographic and sociological characteristics, health literacy, and family health status, have been found to be associated with the extent to which adolescents prioritize medication safety and efficacy when engaging in self-medication practices. Higher levels of health literacy and better family health status were positively correlated with considering both the efficacy and safety of drugs as important factors when self-medicating.

## Introduction

Self-medication is a widespread practice where individuals choose and use drugs to treat self-diagnosed illnesses or symptoms, or the intermittent or continued use of prescribed drugs for chronic or recurrent diseases or symptoms^1^. This phenomenon is widespread among adolescents globally^2–5^. Adolescents frequently opt for over-the-counter (OTC) drugs for self-medication. OTC drugs are easily accessible without a doctor's prescription and are relatively inexpensive, and can be purchased directly from pharmacies^6^.

During adolescence, responsibility for healthcare often shifts from parents and caregivers to the adolescents themselves. As they mature, adolescents typically become more autonomous in managing their medication^7^. A study conducted in 2012 in New Hampshire, USA found that around 90% of adolescents self-medicate with OTC medication by the age of 16^8^. Various factors influence the selection of medication types in self-medication practices among adolescents. These factors impact how adolescents perceive their illnesses and subsequently affect their choice of drugs. Adolescents' characteristics, such as gender, educational level, and socio-economic status, are associated with the frequency of different types of OTC drugs used in self-medication^9^. A 2021 study conducted in Norway found that 33% of adolescent females and 13% of adolescent males used over-the-counter analgesics on a weekly basis^10^.In 2012, a Brazilian study found that girls aged 11 and 15 years^11^ declared to take different types of drugs, especially painkillers^12^, and this behavior has begun to expand to younger ages^13^. In terms of the properties of the drug, the type, efficacy and safety are usually related to the adolescent's choice of self-medication. Studies showed that OTC analgesics^12^, antipyretics^4^, cough suppressants^14^, antibiotics^3^, and anti-inflammatory drugs^15^were widely used among adolescents.

According to reports, OTC drugs containing dextromethorphan hydrobromide (DXM) are frequently misused by adolescents in the United States^16–18^. Acetaminophen, which is found in many OTC cough and cold medications, is also widely used by young people as an analgesic for mild pain or fever^19^. However, adolescents are more likely to misuse OTC medication due to their wrong drug use cognition and limited medication knowledge, thus increasing significant health risks^20^. It is important to note that medication overdoses related to the misuse of common OTC drugs are quite common. A study conducted in Pakistan in 2021 found that adolescents between the ages of 12 and 18 accounted for a high number of emergency department visits due to medication overdoses, with acetaminophen, other nonsteroidal anti-inflammatory drugs (NSAIDs), and cough medicine being the most common culprits^21^. Overdosing on acetaminophen can result from taking a single large dose or repeatedly exceeding the recommended amount, leading to liver damage or hepatic failure^19,22^. Moreover, excessive consumption of the cough suppressant dextromethorphan can cause mental disorders, including hallucinations and delusions, which is another significant reason for its excessive use by some teenagers^23^.

It is clear from the aforementioned observations that adolescents from diverse demographic backgrounds have varying concerns about drug attributes when self-medicating. Similarly, differences in concerns arise when adolescents choose different types of drugs. However, most reports on adolescents’ concerns about the effectiveness and safety of self-medication come from foreign sources. Disparities exist between the sale of OTC medications in the Chinese mainland and other countries. Comprehensive reports detailing adolescents' concerns regarding the efficacy and safety of self-medicated medicines in the Chinese mainland are lacking. This study aims to evaluate the prevalence of self-medication practices among Chinese adolescents and examine the factors they consider when purchasing and using OTC drugs. The language used in this text is clear, concise, and objective, with a formal register and precise word choice. The structure is logical, with causal connections between statements, and the text is free from grammatical errors, spelling mistakes, and punctuation errors. The content of the improved text is as close as possible to the source text, and no new aspects have been added. The anticipated outcomes aim to provide empirical evidence for the formulation of health policies and help to reduce the inappropriate use of OTC drugs among Chinese adolescents.

## Methods

For a detailed description of the method section see our previous study^24,25^.

### Study design

The research data came from China Family Health Index-2021 (CFHI-2021)^26^. The investigation conducted multi-stage sampling in the Chinese mainland. This study was approved by the Medical Ethics Committee of Jinan University (Approval number: JNUKY-2021-018). All participants were informed and willingly signed a consent form prior to their involvement.

### Participants

#### Sample size

In the cross-sectional investigation, the sample size for the bilateral test was determined using Formula 1. The test level was set at α = 0.05. The proportion (p) of adolescents who reported self-medication behavior in Taiwan, China in 2019 was used, which was 33.9%^27^. The allowable error (δ) was set as 0.1. By applying the sample size formula, a minimum sample size of N = 780 was calculated for this study. Taking into account a 15% questionnaire inefficiency rate, the sample size was adjusted to 918. In this study, a total of 1065 questionnaires were collected, exceeding the minimum required sample size (all participants came from the Chinese mainland).

1 $$N=\frac{{Z}_{\alpha /2}^{2}{\text{pq}}}{\delta }$$

#### Inclusion criteria

The inclusion criteria for participants in this study were as follows: (1) Age between 12 and 18 years old. Considering the possibility of respondents providing nominal ages (age filled in being greater than their actual age), 18-year-old participants were included in the study; (2) Self-reported purchase and use of over-the-counter drugs (respondents answered "yes" to the question regarding whether they had purchased and used over-the-counter drugs on their own); (3) Voluntary participation in the study and completion of the informed consent form; (4) Participants could independently complete the online questionnaire or receive assistance from investigators if needed.

#### Exclusion criteria

1. Individuals who are unconscious or have mental disorders.
2. Participants currently involved in other similar research projects.

In this study, a total of 1065 questionnaires were collected and subjected to logic checks. 62 questionnaires exhibiting logical inconsistencies and less than 240 s of filling time were excluded, as were 34 questionnaires with no self-medication behavior. Ultimately, 966 respondents were included, yielding an effective recovery rate of 90.99%.

### Instruments

The questionnaire consists of three parts. The first part investigated the demographic and sociological characteristics of respondents, such as gender, age, ethnicity, location, place of residence (urban and rural), education level, number of siblings, per capita monthly family income(RMB to the USD exchange rate is the average rate for August 2021) etc. The second part investigated the status quo and essential considerations of adolescents' self-medication behavior, consisting of 3 questions (1 single-choice and 2 multiple-choice questions). The third part is a series of standard scales, including the Short Form Health Literacy Instrument (HLS-SF12), Family Health Scale-Short Form (FHS-SF), the Patient Health Questionnaire-9 (PHQ-9) and the General Anxiety Disorder-7(GAD-7).

#### The status quo and important considerations of residents' self-medication behavior

The first single-choice question was "Have you ever purchased and used OTC drugs by yourself?", which was designed to ask whether the respondents had self-medicated. Those who answered "no" were excluded from the study. The first multiple-choice question was, "What kinds of OTC drugs have you purchased and used on your own?" It was designed to investigate the types of OTC drugs the respondents had purchased and used by themselves. The second multiple-choice question was "Which of the following factors do you think are the important factors for you to consider when you purchase OTC drugs by yourself?" to investigate the essential factors for the subjects to purchase OTC drugs by themselves. For each respondent, the order of the two multiple-choice questions was set as random to minimize bias in the study.

#### Health literacy scale short form 12 items

The Health Literacy Scale Short Form 12 Items (HLS-SF12) was used to study the respondents' Health Literacy (HL). The HLS-SF12 scale was originally developed by Duong TV et al. and subsequently translated into Chinese version by Xiaonan Sun et al.^28,29^ The scale includes 3 dimensions of health care, disease prevention and health promotion, with 12 items. Each item is scored at 4 levels (1 = very difficult, 2 = difficult, 3 = easy, 4 = very easy). The standardized HL index is calculated using the formula. The index range is 0–50, and its score positively correlates with the respondents' health literacy. The calculation formula is index = (x − 1) × (50/3), and x is the average score of the items in one dimension or the whole scale of one respondent. 1 is the lowest possible value of the average (at this time, the minimum value of the index is 0), 3 is the range of the average score, and 50 is the maximum value of the index^30^. The higher the index, the higher the health literacy level of the respondents. In this study, the Cronbach's coefficient of HLS-SF12 was 0.937, and the Cronbach's coefficient of health care, disease prevention and health promotion subscales were 0.850, 0.856 and 0.871, respectively, indicating good reliability. Concerning relevant literature, the health literacy of the respondents was divided into the high group (more than 33 points) and the low group (33 points or less). The grouping rules of each subscale were consistent with those of the full scale^31^.

#### Family health scale-short form

The Family Health Scale-Short Form (FHS-SF) was developed by Crandall and Weiss-Laxer et al. and translated into Chinese by Wang Fei et al. according to the standard translation process of the Scale. FHS-SF includes 10 items, 4 dimensions. Each dimension comprises 2–3 items with higher factor loading and weight extracted from the 4 dimensions of the Family Health Scale-long Form(FHS-LF)^32^. The 4 dimensions are Family/social/emotional health processes, Family healthy lifestyle, Family health resources, and external social supports. Each item adopts the Likert 5-level scoring method, among which the sixth, ninth and tenth adopt reverse scoring; the higher the score is, the higher the family health level is^32^. In this study, the Cronbach's coefficient of the FHS-SF scale was 0.851, and the Cronbach's coefficient of the family/social/emotional health process, healthy family lifestyle, family health resources and family external social support subscales were 0.911, 0.874, 0.784 and 0.760, respectively, indicating good reliability. Each dimension of the scale was included in this study for analysis. The respondents were divided into the high group (greater than or equal to the median) and low group (less than the median) according to the score of each dimension of FHS-SF.

#### Mental health status

In this study, the Patient Health Questionision-9(PHQ-9) and the General Anxiety Disorder-7(GAD-7) were used to measure the mental health status of the respondents. The Chinese versions of both scales have been validated, and the Chinese versions still measure depression and anxiety well^33^. In this study, the Cronbach's coefficient of the Patient Health Questionnaire-9 (PHQ-9) and the General Anxiety Disorder-7(GAD-7) were 0.926 and 0.953, respectively, indicating good reliability.

PHQ-9 was developed by Spitzer et al.^34^, which was used to measure the actual feelings of the respondents over nearly two weeks. The Likert four-point scoring method was adopted to evaluate the following 9 aspects: decreased interest, low mood, sleep disturbance, fatigue, eating disorder, inferiority complex, difficulty concentrating, psychomotor delay, and suicidal symptoms. The main statistical index of this scale is the total score, and the total value of PHQ ranges from 0 to 27 points. The higher the score, the higher the degree of depression. In this study, according to the scoring rules, the respondents were divided into two groups: no depression (4 points or less) and possible depression (more than 4 points).

GAD-7 was developed by Spitzer et al. (2006)^35^ to measure the degree of anxiety. It uses the number of days with related symptoms in the last two weeks as the evaluation standard. The degree of tension and anxiety, uncontrollable worry, excessive worry, inability to relax, inability to sit still, irritability, anger, and fear were respectively assessed. The main statistical index of this scale is the total score, which is scored by Likert four points. The total score ranges from 0 to 21, mainly used to assess the severity of anxiety symptoms; the higher the score, the higher the degree of anxiety. In this study, according to the scoring rules, the respondents were divided into two groups: no anxiety (score 4 or less) and possible anxiety (score more than 4).

#### Statistical analysis

Statistical analysis was performed using SPSS 25.0 (SPSS, Inc., Chicago, IL, USA Network Version from Peking University, Address: 162.105.134.153) and R4.3.2. In this study, all scale scores were transformed into binary variables (high and low groups) by referring to relevant literature^36^. The classification variables were represented by frequency (component ratio), and univariate binary logistic regression was used for univariate analysis. Multivariate binary stepwise logistic regression was used to analyze the factors related to the consideration of drug efficacy and safety among respondents. The inclusion criteria for variables was α = 0.05, while the exclusion criteria was α = 0.10. The test level was set at α = 0.05. Finally, the respondents were divided into different subgroups based on gender and permanent residence, and multivariate binary stepwise logistic regression was conducted for each subgroup to perform a subgroup analysis. The Chinese map was drawn using the R/ggplot2 package with the open source data set (https://datav.aliyun.com/portal/school/atlas/area_selector).

### Quality control

Two rounds of preliminary investigations were conducted before the formal investigation to refine the questionnaire based on the results. Trained investigators distributed questionnaires and assigned unique codes to each respondent to ensure anonymity. Every Sunday night, the research staff reviewed and provided feedback on the questionnaires collected by the investigators. After the questionnaire was collected, the logic check and data screening were carried out by two people back to back. If an outlier was identified during data analysis, the research team cross-checked the original questionnaire with the investigator before proceeding with further analysis.

### Ethical approval and consent to participate

The study was conducted in accordance with the ethical standards set forth in the Helsinki Declaration (1983). This study was approved by the Medical Ethics Committee of Jinan University (Approval number: JNUKY-2021-018). All respondents in this study had a thorough understanding of the contents outlined in the informed consent form, and only those who voluntarily agreed to participate by signing the form were included.

## Results

### Harman’s single factor test

This study examined common method bias using the Harman’s single-factor test. The results showed that the number of factors with a characteristic root greater than one was five. The variance contribution of the first principal factor was 32.09%, which did not exceed 40%, indicating that there was no obvious common method bias.

### Demographic and sociological characteristics of respondents and important considerations in their self-medication behaviors

There were a few more female respondents than male respondents in this study; the largest number of respondents were from eastern China, and most of the respondents' usual place of residence was urban. The demographic and sociological characteristics are shown in Table 1.

**Table 1:** General demographic characteristics of participants (N = 969).

Variable	Number	Percentage
Location		
Eastern China	464	47.88%
Middle China	270	27.86%
Western China	235	24.25%
Place of residence		
Urban	694	71.62%
Rural	275	28.38%
Monthly income^a^		
￥0–4500	525	54.18%
￥4501–15,000	407	42.00%
≥ ￥15,001	37	3.82%
Gender		
Male	403	41.59%
Female	566	58.41%
Ethnicity		
Han	893	92.16%
Minorities	76	7.84%
Education level		
Undergoing higher education	327	33.75%
Not in higher education	642	66.25%
Only child		
Yes	310	31.99%
No	659	68.01%

^a^￥4500(equal to$696.59),￥4501(equal to$696.75),￥15,000(equal to$2321.98),￥15,001(equal to$2322.14).

**Figure 1:**
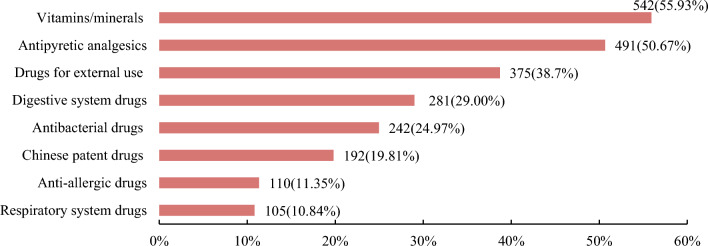
Types of OTC medicines that respondents bought and used on their own.

The findings showed that 96.61% (969/1003) of Chinese 12–18 years old had self-medication behaviors, and the top two types of OTC drugs purchased and used by 12–18 years old were vitamins/minerals (542, 55.93%) and antipyretics and analgesics (491, 50.67%). In this study, self-purchase and use of gynecological medication were included only for female respondents. Because only women tend to buy these drugs, the purchase of these drugs by men was not analyzed in this study. The percentage of respondents who had purchased and used gynecological drugs on their own was 4.33% of all female respondents. Of the 22 people who chose "other" and filled in the blanks, 12 filled in "anti-cold medicine", 2 filled in "ibuprofen", 2 filled in "digestive medicine", "anti-inflammatory", broad-spectrum antibiotic "Amoxicillin", eye drops and Niuhuang antidote tablets were each filled by one person, as shown in Fig. 1.

**Figure 2:**
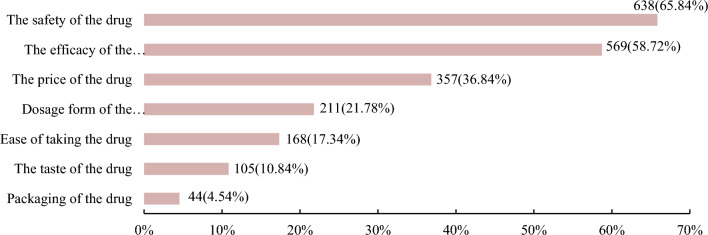
Factors of the drug itself that respondents focus on when self-medicating.

The top two important considerations when purchasing OTC medicines were the safety of the medicine (638 participants, 65.84%) and the efficacy of the medicine (569 participants, 58.72%), as shown in Fig. 2.

**Figure 3:**
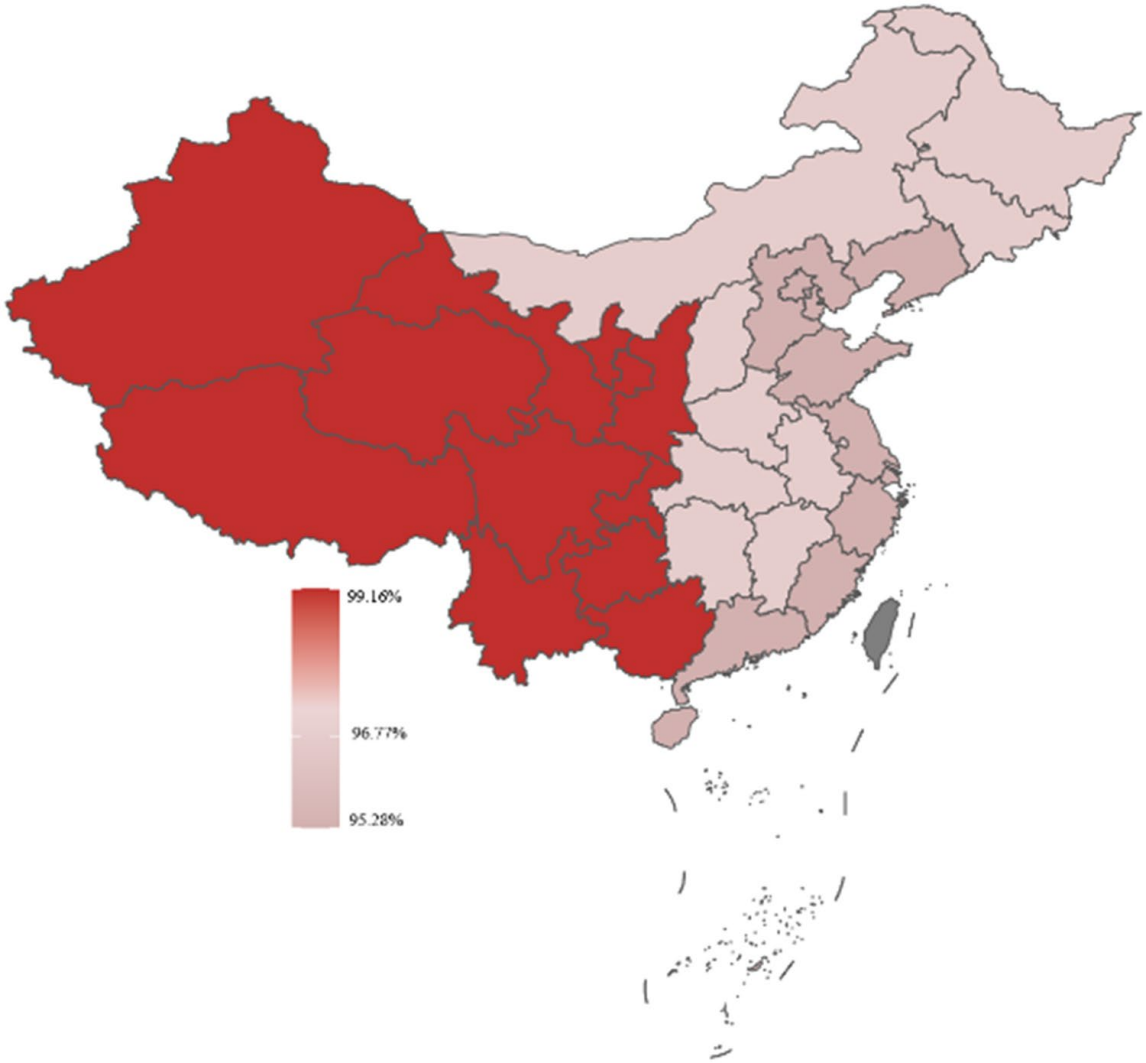
Distribution of self-medication behaviors by region.

**Figure 4:**
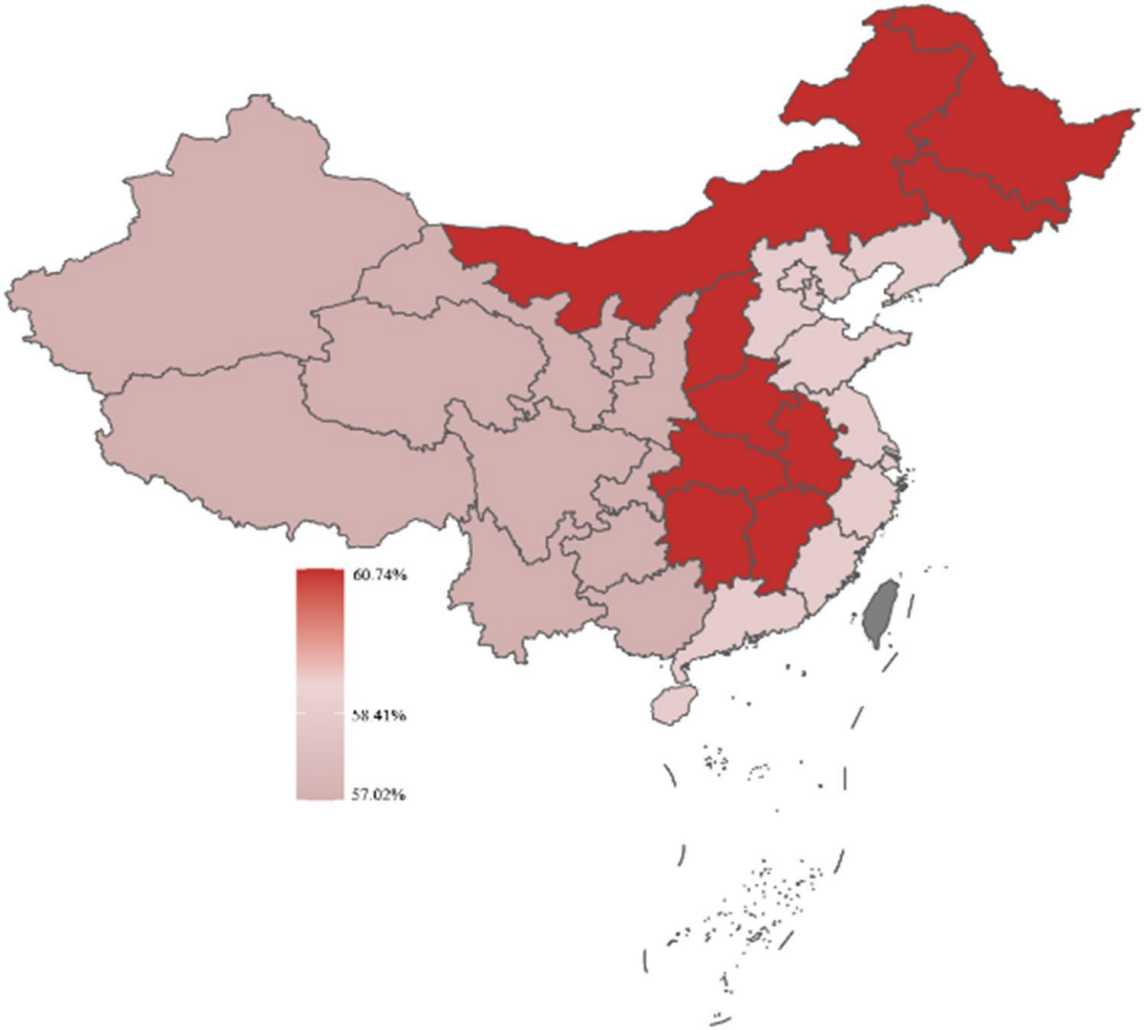
The consideration of drug effectiveness by individuals engaging in self-medication exhibited variation across diverse areas.

**Figure 5:**
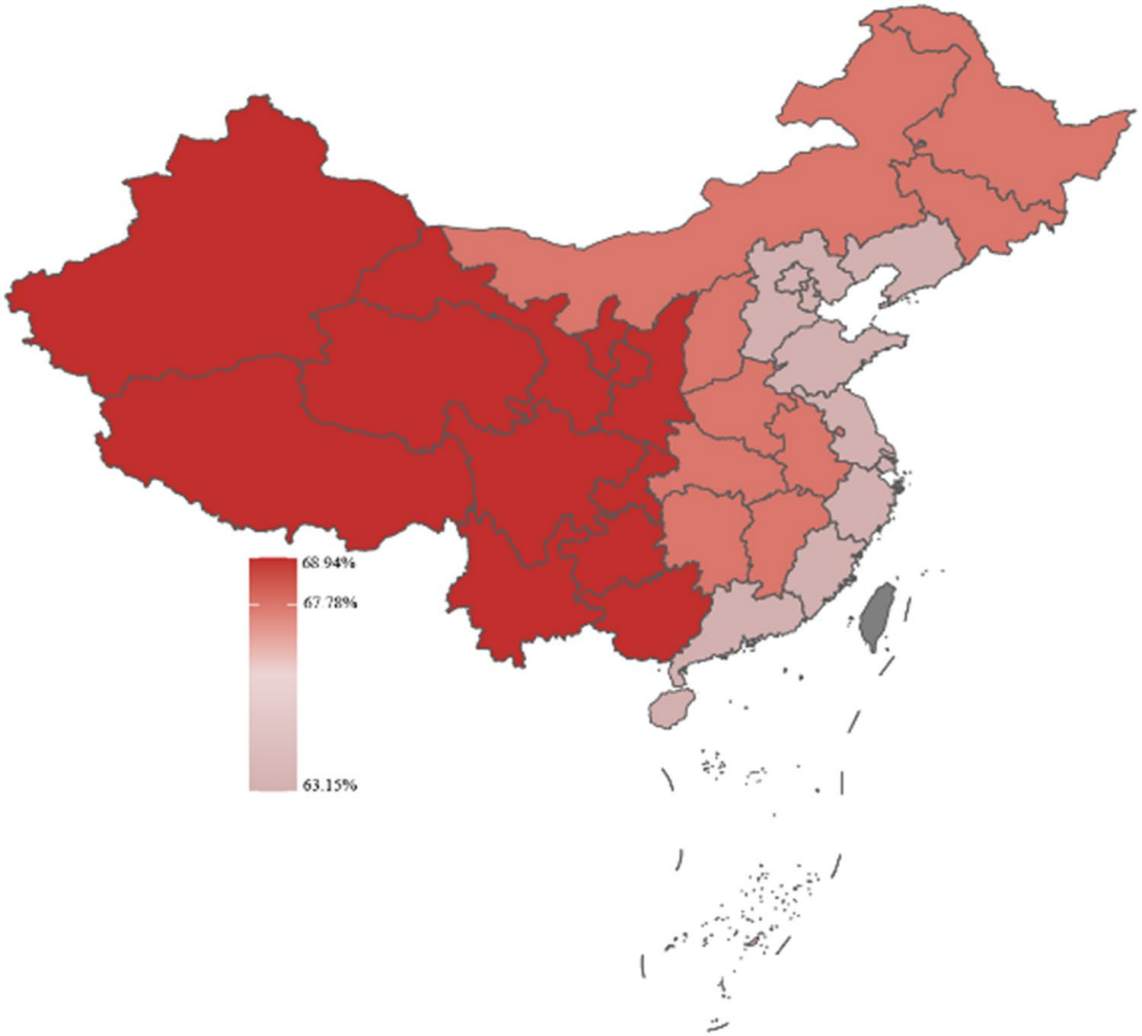
The consideration of drug safety by individuals engaging in self-medication exhibited variation across diverse areas.

Respondents in this study were categorized as being from eastern, central, and western China based on their location. According to the geographic heat map, respondents from western China had the highest prevalence of self-medication, more respondents from central China considered the efficacy of OTC drugs when self-medication, and more respondents from western China considered the safety of OTC drugs when self-medication. For details, in Figs. 3, 4, 5.

### Participant scores on the scales

The scores of HLS-SF12, FHS-SF, PHQ-9 and GAD-7 are shown in Table 2. As none of the scale scores met a normal distribution, the median, upper and lower quartiles were used to describe the concentration and dispersion degree trends of each scale score. The details of the scale scores for each scale are shown in Table 2.

**Table 2:** Scores on each scale.

	Appraisal Content	No. of items	Score range	Kolmogorow-Smironov Z	P value from K–S test	Median	Q1–Q3	NO. and percentage of high score group	NO. and percentage of low score group
HLS-SF12	Total scale	12	0–50	0.187	< 0.001	33.33	31.94–38.89	637(69.45%)	296(30.55%)
	Healthcare	4	0–50	0.221	< 0.001	33.33	29.17–41.67	723(74.61%)	246(25.39%)
	Disease prevention	4	0–50	0.252	< 0.001	33.33	33.33–41.67	730(75.34%)	239(24.66%)
	Health promotion	4	0–50	0.258	< 0.001	33.33	33.33–41.67	780(80.50%)	189(19.50%)
FHS-SF	Total scale	10	20–50	0.066	< 0.001	39.00	34.00–44.00	701(72.34%)	268(27.66%)
	Family/social/emotional health process	3	3–15	0.162	< 0.001	12.00	10.00–14.00	625(64.50%)	344(35.50%)
	Family healthy lifestyle	2	2–10	0.186	< 0.001	8.00	7.00–10.00	675(69.66%)	294(30.34%)
	Family health resources	3	3–15	0.106	< 0.001	11.00	9.00–14.00	567(58.51%)	402(41.49%)
	External family social support	2	2–10	0.187	< 0.001	8.00	6.00–9.00	594(61.30%)	375(38.70%)
PHQ-9	Depressive tendencies	9	0–27	0.138	< 0.001	5.00	1.00–9.00	393(40.56%)	576(59.44%)
GAD-7	Anxiety tendencies	7	0–21	0.182	< 0.001	3.00	0.00–7.00	525(54.18%)	444(45.82%)

### Univariate binary logistic regression analysis of the types of OTC drugs purchased by participants

Univariate binary logistic regression analysis was used to conduct a univariate analysis of the types of OTC drugs the participants had purchased and used on their own. The differences in whether the participants had purchased and used OTC antipyretic and analgesic drugs by location, ethnicity, and education level were statistically significant (*P* < 0.05). Statistically significant differences (*P* < 0.05) were found in whether participants with different education level and family health status had purchased and used antimicrobial OTC drugs independently. Statistically significant differences (*P* < 0.05) were found between participants with different place of residence, gender, family health status, and anxiety status on whether they had purchased and used OTC Chinese patent medicine on their own. See Table 3 for details.

**Table 3:** Univariate binary logistic regression of the types of OTC respondents had purchased.

Drug type	Variable	β	SE	*P*	OR	95%CI
**Antipyretics and analgesics**						
	**Location (control group = Eastern)**					
	Middle	− 0.276	0.153	0.072	0.759	0.562–1.025
	Western	− **0.464**	**0.161**	**0.004**	**0.628**	**0.458–0.862**
	**Place of residence (control group = Rural)**					
	Urban	− 0.258	0.143	0.072	0.773	0.584–1.023
	**Monthly income (RMB) (control group = **$\leqslant$**￥4500)**					
	$\geqslant$ ￥4501	0.150	0.129	0.245	1.162	0.902–1.496
	**Gender (control group = Female)**					
	Male	− 0.020	0.130	0.875	0.980	0.759–1.265
	**Ethnicity (control group = Han)**					
	Minorities	− **0.552**	**0.245**	**0.024**	**0.576**	**0.356–0.931**
	**Education level (control group = Not in higher education)**					
	Undergoing higher education	**0.377**	**0.137**	**0.006**	**1.458**	**1.115–1.906**
	**Single-child (control group = No)**					
	Yes	0.169	0.138	0.219	1.185	0.904–1.553
	**Health literacy (control group = Low score group)**					
	High score group	− 0.117	0.140	0.402	0.889	0.677–1.169
	**Family health (control group = Low score group)**					
	High score group	− 0.045	0.144	0.752	0.956	0.721–1.266
	**Depression (control group = No)**					
	Yes	− 0.034	0.129	0.794	0.967	0.751–1.245
	**Anxiety (control group = No)**					
	Yes	− 0.002	0.131	0.986	0.998	0.772–1.289
**Antibacterial drugs**						
	**Location (control group = Eastern)**					
	Middle	− 0.013	0.176	0.941	0.987	0.699–1.394
	Western	− 0.086	0.187	0.644	0.917	0.636–1.322
	**Place of residence (control group = Rural)**					
	Urban	− 0.213	0.169	0.207	1.238	0.889–1.724
	**Monthly income (RMB) (control group = **$\leqslant$**￥4500)**					
	$\geqslant$ ￥4501	0.025	0.149	0.868	1.025	0.766–1.372
	**Gender (control group = Female)**					
	Male	− 0.291	0.153	0.057	0.747	0.554–1.009
	**Ethnicity (control group = Han)**					
	Minorities	0.149	0.268	0.577	1.161	0.687–1.963
	**Education level (control group = Not in higher education)**					
	Undergoing higher education	**0.368**	**0.154**	**0.016**	**1.446**	**1.070–1.953**
	**Single-child (control group = No)**					
	Yes	0.213	0.156	0.173	1.238	0.911–1.682
	**Health literacy (control group = Low score group)**					
	High score group	0.156	0.164	0.340	1.169	0.848–1.612
	**Family health (control group = Low score group)**					
	High score group	**0.343**	**0.174**	**0.048**	**1.409**	**1.002–1.980**
	**Depression (control group = No)**					
	Yes	0.109	0.149	0.467	1.115	0.832–1.494
	**Anxiety (control group = No)**					
	Yes	− 0.049	0.152	0.745	0.925	0.707–1.281
**Chinese patent drugs**						
	**Location (control group = Eastern)**					
	Middle	− 0.165	0.192	0.390	0.848	0.582–1.235
	Western	− 0.262	0.206	0.202	0.769	0.514–1.151
	**Place of residence (control group = Rural)**					
	Urban	**0.576**	**0.197**	**0.003**	**1.779**	**1.209–2.618**
	**Monthly income (RMB) (control group = **$\leqslant$**￥4500)**					
	$\geqslant$ ￥4501	0.313	0.162	0.052	1.368	0.997–1.878
	**Gender (control group = Female)**					
	Male	− **0.437**	**0.169**	**0.010**	**0.646**	**0.464–0.900**
	**Ethnicity (control group = Han)**					
	Minorities	− 0.005	0.300	0.986	0.995	0.552–1.791
	**Education level (control group = Not in higher education)**					
	Undergoing higher education	0.121	0.169	0.473	1.128	0.811–1.570
	**Single-child (control group = No)**					
	Yes	0.307	0.168	0.068	1.359	0.977–1.889
	**Health literacy (control group = Low score group)**					
	High score group	0.051	0.176	0.773	1.052	0.745–1.486
	**Family health (control group = Low score group)**					
	High score group	**0.694**	**0.205**	**0.001**	**2.003**	**1.341–2.990**
	**Depression (control group = No)**					
	Yes	− 0.131	0.161	0.417	0.877	0.639–1.203
	**Anxiety (control group = No)**					
	Yes	− **0.356**	**0.169**	**0.035**	**0.701**	**0.503–0.976**
**Digestive system drugs**						
	**Location (control group = Eastern)**					
	Middle	− 0.196	0.169	0.248	0.822	0.590–1.146
	Western	− 0.270	0.179	0.132	0.764	0.537–1.085
	**Place of residence (control group = Rural)**					
	Urban	0.068	0.158	0.666	1.071	0.785–1.459
	**Monthly income (RMB) (control group = **$\leqslant$**￥4500)**					
	$\geqslant$ ￥4501	− 0.116	0.142	0.414	0.890	0.673–1.177
	**Gender (control group = Female)**					
	Male	− 0.142	0.145	0.324	0.867	0.653–1.151
	**Ethnicity (control group = Han)**					
	Minorities	**0.512**	**0.246**	**0.038**	**1.668**	**1.030–2.703**
	**Education level (control group = Not in higher education)**					
	Undergoing higher education	**0.312**	**0.147**	**0.034**	**1.366**	**1.024–1.824**
	**Single-child (control group = No)**					
	Yes	0.207	0.150	0.167	1.230	0.917–1.649
	**Health literacy (control group = Low score group)**					
	High score group	0.091	0.155	0.555	1.096	0.809–1.484
	**Family health (control group = Low score group)**					
	High score group	− 0.204	0.156	0.190	0.816	0.601–1.106
	**Depression (control group = No)**					
	Yes	**0.446**	**0.145**	**0.002**	**1.561**	**1.176–2.073**
	**Anxiety (control group = No)**					
	Yes	**0.330**	**0.143**	**0.021**	**1.391**	**1.051–1.842**
**Respiratory system drugs**						
	**Location (control group = Eastern)**					
	Middle	− 0.486	0.258	0.059	0.615	0.371–1.019
	Western	− 0.433	0.267	0.104	0.648	0.384–1.094
	**Place of residence (control group = Rural)**					
	Urban	0.323	0.244	0.185	1.382	0.856–2.230
	**Monthly income (RMB) (control group = **$\leqslant$**￥4500)**					
	$\geqslant$ ￥4501	0.167	0.207	0.420	1.181	0.788–1.772
	**Gender (control group = Female)**					
	Male	0.102	0.208	0.625	1.107	0.736–1.666
	**Ethnicity (control group = Han)**					
	Minorities	0.109	0.371	0.769	1.115	0.539–2.308
	**Education level (control group = Not in higher education)**					
	Undergoing higher education	− 0.118	0.222	0.595	0.889	0.575–1.373
	**Single-child (control group = No)**					
	Yes	0.257	0.215	0.232	1.293	0.849–1.970
	**Health literacy (control group = Low score group)**					
	High score group	− **0.471**	**0.213**	**0.027**	**0.625**	**0.412–0.948**
	**Family health (control group = Low score group)**					
	High score group	**− 0.587**	**0.214**	**0.006**	**0.556**	**0.365–0.847**
	**Depression (control group = No)**					
	Yes	0.356	0.212	0.094	1.427	0.942–2.163
	**Anxiety (control group = No)**					
	Yes	**0.581**	**0.208**	**0.005**	**1.787**	**1.190–2.685**
**Vitamins/minerals**						
	**Location (control group = Eastern)**					
	Middle	0.113	0.154	0.464	1.119	0.828–1.514
	Western	0.215	0.162	0.186	1.239	0.902–1.703
	**Place of residence (control group = Rural)**					
	Urban	**0.633**	**0.144**	** < 0.001**	**1.883**	**1.420–2.497**
	**Monthly income (RMB) (control group = **$\leqslant$**￥4500)**					
	$\geqslant$ ￥4501	**0.418**	**0.131**	**0.001**	**1.519**	**1.176–1.964**
	**Gender (control group = Female)**					
	Male	− **0.386**	**0.131**	**0.003**	**0.680**	**0.525–0.879**
	**Ethnicity (control group = Han)**					
	Minorities	− 0.259	0.239	0.279	0.772	0.483–1.233
	**Education level (control group = Not in higher education)**					
	Undergoing higher education	**0.323**	**0.138**	**0.019**	**1.381**	**1.053–1.812**
	**Single-child (control group = No)**					
	Yes	0.147	0.139	0.292	1.158	0.882–1.522
	**Health literacy (control group = Low score group)**					
	High score group	**0.365**	**0.140**	**0.009**	**1.440**	**1.094–1.895**
	**Family health (control group = Low score group)**					
	High score group	**0.750**	**0.146**	** < 0.001**	**2.118**	**1.591–2.819**
	**Depression (control group = No)**					
	Yes	− 0.045	0.130	0.730	0.956	0.741–1.233
	**Anxiety (control group = No)**					
	Yes	− 0.222	0.132	0.091	0.801	0.618–1.036
**External skin drugs**						
	**Location (control group = Eastern)**					
	Middle	0.084	0.157	0.595	1.087	0.799–1.480
	Western	0.150	0.164	0.360	1.162	0.843–1.601
	**Place of residence (control group = Rural)**					
	Urban	0.160	0.148	0.278	1.174	0.879–1.568
	**Monthly income (RMB) (control group = **$\leqslant$**￥4500)**					
	$\geqslant$ ￥4501	− 0.032	0.132	0.809	0.968	0.747–1.255
	**Gender (control group = Female)**					
	Male	− **0.361**	**0.135**	**0.008**	**0.697**	**0.534–0.909**
	**Ethnicity (control group = Han)**					
	Minorities	− 0.147	0.249	0.554	0.863	0.529–1.407
	**Education level (control group = Not in higher education)**					
	Undergoing higher education	0.241	0.139	0.083	1.272	0.969–1.669
	**Single-child (control group = No)**					
	Yes	− 0.080	0.142	0.575	0.923	0.699–1.220
	**Health literacy (control group = Low score group)**					
	High score group	0.114	0.144	0.427	1.121	0.846–1.487
	**Family health (control group = Low score group)**					
	High score group	**0.395**	**0.152**	**0.009**	**1.484**	**1.103–1.998**
	**Depression (control group = No)**					
	Yes	0.085	0.133	0.523	1.088	0.839–1.411
	**Anxiety (control group = No)**					
	Yes	− 0.165	0.135	0.222	0.848	0.651–1.105
**Gynecological drugs**^a^						
	**Location (control group = Eastern)**					
	Middle	− 0.096	0.421	0.820	0.908	0.398–2.075
	Western	− 0.133	0.438	0.761	0.875	0.371–2.066
	**Place of residence (control group = Rural)**					
	Urban	− 0.247	0.377	0.513	0.781	0.373–1.636
	**Monthly income (RMB) (control group = **$\leqslant$**￥4500)**					
	$\geqslant$ ￥4501	0.549	0.353	0.119	1.732	0.868–3.456
	**Ethnicity (control group = Han)**					
	Minorities	0.037	0.624	0.953	1.038	0.305–3.525
	**Education level (control group = Not in higher education)**					
	Undergoing higher education	**1.030**	**0.361**	**0.004**	**2.801**	**1.380–5.684**
	**Single-child (control group = No)**					
	Yes	0.008	0.376	0.984	1.008	0.482–2.106
	**Health literacy (control group = Low score group)**					
	High score group	**− 0.751**	**0.353**	**0.033**	**0.472**	**0.236–0.942**
	**Family health (control group = Low score group)**					
	High score group	0.726	0.493	0.141	2.066	0.786–5.431
	**Depression (control group = No)**					
	Yes	0.405	0.367	0.270	1.499	0.730–3.075
	**Anxiety (control group = No)**					
	Yes	0.626	0.353	0.076	1.870	0.937–3.734
**Antiallergic drugs**						
	**Location (control group = Eastern)**					
	Middle	**− **0.401	0.255	0.116	0.670	0.406–1.104
	Western	**− **0.115	0.248	0.641	0.891	0.549–1.447
	**Place of residence (control group = Rural)**					
	Urban	**0.513**	**0.250**	**0.040**	**1.670**	**1.023–2.725**
	**Monthly income (RMB)**^**a**^** (control group = **$\leqslant$**￥4500)**					
	$\geqslant$ ￥4501	0.148	0.203	0.465	1.160	0.779–1.725
	**Gender (control group = Female)**					
	Male	**− 0.521**	**0.218**	**0.017**	**0.594**	**0.388–0.910**
	**Ethnicity (control group = Han)**					
	Minorities	**0.725**	**0.308**	**0.018**	**2.066**	**1.130–3.777**
	**Education level (control group = Not in higher education)**					
	Undergoing higher education	0.130	0.211	0.538	1.139	0.753–1.722
	**Single-child (control group = No)**					
	Yes	0.220	0.211	0.297	1.247	0.824–1.886
	**Health literacy (control group = Low score group)**					
	High score group	0.128	0.224	0.568	1.137	0.732–1.765
	**Family health (control group = Low score group)**					
	High score group	0.352	0.243	0.148	1.422	0.883–2.289
	**Depression (control group = No)**					
	Yes	0.397	0..209	0.057	1.487	0.988–2.239
	**Anxiety (control group = No)**					
	Yes	0.143	0.204	0.485	1.153	0.773–1.722

^a^Only female participants were included.

Significant values are in bold.

### Univariate binary logistic regression of participants regard drug efficacy and safety as important considerations in purchasing OTC

Univariate binary logistic regression analysis of the variables related to the efficacy and safety of the drugs considered by the respondents when purchasing OTC drugs was conducted. Differences in whether participants with different health care scores in health literacy, different family/social/emotional health process scores in family health, different family health lifestyle scores, different family health resource scores, and different anxiety status focused on the factor of drug efficacy when purchasing OTC medications were statistically significant (*P* < 0.05). Statistically significant differences (*P* < 0.05) were found between respondents with different monthly per capita household income, different disease prevention scores in health literacy, different family/social/emotional health process scores in family health, different family health lifestyle scores, and different family health resource scores on the factor of whether they focused on drug safety when purchasing OTC. See supplementary Materials, S2 Table for details.

### Multifactor analysis of whether the efficacy or safety of drugs is an important consideration for respondents purchasing OTC drugs by themselves

#### Drug efficacy

In this study, the dependent variable was whether respondents considered the drug efficacy as an important factor in self-medication. The regression model included location, place of residence, and gender as mandatory demographic characteristics. Additionally, monthly household income, ethnicity, education, being an only child, health literacy, family health status, and depression and anxiety were included in the regression model using stepwise regression. In particular, health literacy and family health were included in the model by calculating the total score and also by scoring the different dimensions of the scale measures.

The Omnibus test result of the established model is *P* < 0.05, the − 2 log-likelihood value is 1292.025, and the Hosmer-Lameshaw test result is *P* = 0.467 > 0.05, indicating that the model is of good quality. Logistic regression analysis indicated that health care literacy and family healthy lifestyle was associated with whether respondents considered drug efficacy as an essential consideration when purchasing OTC. Compared to those with lower health care literacy, those with better health care literacy are more likely to consider drug efficacy as an essential factor(OR = 1.537, 95%CI 1.142–2.070 *P* < 0.05). The high family healthy lifestyle score group was more likely to cite efficacy as an important factor than the low family healthy lifestyle score group (OR = 1.476, 95%CI 1.113–1.956 *P* < 0.05). See Table 4 for details.

**Table 4:** Multivariate binary stepwise logistic regression results: whether respondents consider drug efficacy as an important consideration in purchasing OTC.

Variable	β	SE	*P*	OR	95%CI
Location (control group = Eastern)					
Middle	0.081	0.158	0.608	1.085	0.795–1.479
Western	− 0.083	0.164	0.611	0.920	0.667–1.269
Place of residence (control group = Rural)					
Urban	0.200	0.146	0.172	1.221	0.917–1.627
Gender (control group = Female)					
Male	0.045	0.134	0.740	1.046	0.803–1.361
Health care (control group = Low score group)					
High score group	**0.430**	**0.152**	**0.005**	**1.537**	**1.142–2.070**
Family Healthy Lifestyle (control group = Low score group)					
High score group	**0.389**	**0.144**	**0.047**	**1.476**	**1.113–1.956**

Significant values are in bold.

#### Drug safety

In this study, the dependent variable was whether respondents considered the drug safety as an important factor in self-medication. The regression model included location, place of residence, and gender as mandatory demographic characteristics. Additionally, monthly household income, ethnicity, education, being an only child, health literacy, family health status, and depression and anxiety were included in the regression model using stepwise regression. In particular, health literacy and family health were included in the model by calculating the total score and also by scoring the different dimensions of the scale measures.

The Omnibus test result of the established model is *P* < 0.05, the − 2 log-likelihood value is 1183.733, and the Hosmer-Lameshaw test result is *P* = 0.483 > 0.05, indicating that the model is of good quality. Logistic regression analysis indicated that place of residence, monthly per capita household income, whether only child, disease prevention literacy, and household health resources were associated with whether respondents considered drug safety as an essential consideration when purchasing OTC. Participants with higher monthly per capita household income (≥ ￥4,501≈$696.75) are less likely to consider safety as an essential factor than those with lower monthly per capita household income (≤ ￥4,500≈$696.59)(OR = 0.532, 95%CI 0.398–0.713 *P* < 0.001); participants who were sole offspring were also found to exhibit a lower inclination, compared to their counterparts with siblings, towards prioritizing the safety aspect of self-medication practices. (OR = 0.727, 95CI% 0.536–0.987 *P* < 0.05). Participants residing in urban areas exhibited a higher propensity to prioritize the safety of the drug itself when engaging in self-medication, in comparison to their counterparts residing in rural areas(OR = 1.454, 95CI% 1.054–2.005 *P* < 0.05); compared to respondents with lower disease prevention scores, respondents with higher disease prevention scores were more likely to consider drug safety as an essential factor(OR = 1.544, 95CI% 1.129–2.111 *P* < 0.05); The safety of medications was more likely to be an essential consideration for those with better family health resources in their family health status than for those with worse family health resources in their family health status(OR = 2.180, 95%CI 1.643–2.892 *P* < 0.001), For details, see Table 5.

**Table 5:** Multivariate binary stepwise logistic regression results: whether respondents consider drug safety as an important consideration in purchasing OTC.

Variable	β	SE	*P*	OR	95%CI
Location (control group = Eastern)					
Middle	0.072	0.170	0.673	1.074	0.771–1.498
Western	0.220	0.176	0.211	1.246	0.883–1.760
Place of residence (control group = Rural)					
Urban	**0.374**	**0.164**	**0.022**	**1.454**	**1.054–2.005**
Gender (control group = Female)					
Male	− 0.051	0.142	0.719	0.950	0.719–1.225
Monthly income (RMB)^a^ (control group = < ￥4500)					
≥ ￥4501	− **0.630**	**0.149**	** < 0.001**	**0.532**	**0.398–0.713**
Single-child (control group = No)					
Yes	− **0.319**	**0.156**	**0.041**	**0.727**	**0.536–0.987**
Disease Prevention (control group = Low score group)					
High score group	**0.434**	**0.160**	**0.007**	**1.544**	**1.129–2.111**
Family Health Resources (control group = Low score group)					
High score group	**0.779**	**0.144**	** < 0.001**	**2.180**	**1.643–2.892**

^a^￥4500(equal to$696.59),￥4501(equal to$696.75),￥15,000(equal to$2321.98),￥150,001(equal to$2322.14).

Significant values are in bold.

Subgroup analysis was used with subgroup classification based on gender, location and place of residence to explore the variability of factors related to the dependent variable among different subgroups. The study results showed that the model built using data from each subgroup was generally consistent with the total model. See Supplementary Materials, S4–S8 Table for details.

## Discussion

### Chinese adolescents’ self-medication behaviors

Currently, nearly half of the people in the world do not have access to basic health services. Self-care has become a crucial way to promote primary health care and protect health for all. Thus, self-medication is widely used by people around the world as an important means of self-care. Studies have shown that self-medication is common among adolescents in countries including China, Australia, Malaysia, etc.^37,38^. This study reveals that the self-medication rate among Chinese adolescents aged 12–18 years is alarmingly high at 96.61%, suggesting that a majority of Chinese adolescents in this age range engage in self-medication.

Among all kinds of OTC drugs, the respondents who bought vitamins/minerals by themselves account for the most significant proportion. This indicates that most adolescents require vitamin/mineral supplementation. This phenomenon may be related to the fact that they are in a critical period of growth. At this stage, they are influenced by external factors such as health education at school and promotion by pharmaceutical manufacturers to take vitamins/minerals in order to grow taller or reduce the occurrence of limb cramps. Previous studies^39,40^ have shown that most adolescents face various kinds of vitamin and mineral deficiencies due to factors such as body growth, diet, exercise, and so on. The proportion of respondents who purchased antipyretic and analgesic drugs ranked second, indicating that most adolescents buy OTC drugs to alleviate fever or pain (such as dysmenorrhea, headache, muscle pain, joint pain, etc.). A Portuguese study also revealed that headache was the most common issue leading to self-medication among adolescents^41^. Of the 22 who answered "others," 12 responded with "anti-cold medicine," suggesting that some adolescents self-medicate to treat upper respiratory tract infections and the flu. Similar to this finding, the Portuguese study also revealed that the presence of symptoms of upper respiratory infections was a reason for adolescents to engage in self-medication^41^.

### Factors related to drug efficacy or safety as an important consideration in Chinese adolescents

#### Proportion of important considerations when self-medication

The results showed that Chinese adolescents aged 12–18 years pay more attention to the safety, efficacy, and price of OTC drugs when making purchases. However, they give less consideration to characteristics such as drug taste and exquisite packaging. This finding aligns with the research results of Xu Jing et al.^42^, which suggest that adolescents aged 12–18 prioritize the practicality of drugs when choosing and buying OTC drugs, rather than focusing on attributes unrelated to the purpose of the drugs, such as packaging.

#### Drug efficacy

The results of univariate logistic regression analysis showed that the score of health care on the health literacy scale and the score on the family health scale were significantly related to the consideration of drug efficacy. Multivariate stepwise logistic regression analysis further found that better healthcare literacy would improve people's attention to the efficacy of drugs. Health literacy refers to an individual's ability to discover, understand, evaluate, and apply health-related information^43^. In the HLS-SF12 scale, the items in the healthcare dimension mainly involved disease treatment and drug-related information^27^. Therefore, people with higher scores in the healthcare dimension tend to be more aware of drug information and better judge the advantages and disadvantages of treatment options, paying more attention to the therapeutic effect of drugs. In addition, those with high family healthy lifestyle scores were more likely to consider drug efficacy an essential factor. Family is the most basic unit of social life, and a healthy lifestyle manifests good family function. It helps family members to actively understand the knowledge and skills related to drug treatment and change their cognition and behavior to maintain a healthy state^44,45^. Therefore, people with high family healthy lifestyle scores usually have a strong health awareness and pay more attention to the efficacy of drugs.

#### Drug safety

The results of univariate logistic regression analysis showed that the family income per capita, the score of the disease prevention dimension in the health literacy scale and the score of the family health scale were significantly related to the emphasis on drug safety. Multivariate stepwise logistic regression analysis further found that compared with those with higher per capita monthly household income, those with lower per capita monthly household income were more likely to regard drug safety as an essential factor. Sun Shan once pointed out that groups with different economic conditions have apparent differences in OTC drug consumption and expenditure patterns^46^. The lower income group is obliged to receive the drug treatment generally after the disease when pain has affected the everyday work and life of the case^46^. Therefore, they usually pay more attention to the safety of drugs and hope to avoid secondary drug treatment expenses and secondary physical injuries caused by adverse drug reactions or side effects. The results of this study indicated that urban adolescents were more concerned about the safety of medications than rural adolescents. This difference is likely to be influenced by the disparity in education between urban and rural areas. Existing studies suggest that rural adolescents do not improve their health literacy as much as urban adolescents do, even with the same level of health education^47^. Therefore, this group does not possess the necessary knowledge to prioritize medication safety when self-medication. The findings of this study also suggest that non-only children are more likely to be concerned about the safety of medicines when self-medication. This is likely related to the fact that non-only children often need to share the family stockpile of medicines with their siblings. In such cases, non-only children will be exposed to a wider variety of medicines, and therefore, they need to be more concerned about the safety of medicines when self-medication.

At the same time, the higher the score for disease prevention, the more inclined people will be to consider drug safety as an essential factor. In the HLS-SF12 scale, the items of the disease prevention dimension mainly involve emotion management, physical examination cognition and vaccine demand. Therefore, people with higher scores in the disease prevention dimension are more effective in protecting themselves from disease. As a result, they will naturally pay more attention to the drug's safety and carefully judge whether the drug will cause other diseases or adverse reactions. Regarding family health status, those with better family health resources were more willing to consider drug safety as an essential factor. Household health resources refer to the material and non-material assets a household can carry out daily activities and perform its functions, including money, housing, and health care^48,49^. Therefore, those with better family health resources have better access to healthcare resources and are more likely to obtain medical care and scientific medication knowledge, so they pay more attention to drug safety.

### Suggestion

Self-medication plays an integral role in complementing health care and treating diseases. Especially under the influence of the COVID-19 pandemic, many Chinese adolescents who have just entered university need to comply with the epidemic prevention and control regulations of their schools, which may inconvenience their medical treatment. Scientific and rational self-medication can make adolescents get treatment at a lower cost and a higher convenience. However, adolescents' inappropriate self-medication behavior may negatively affect their health. Adolescents' judgment and self-care abilities are typically far worse than adults. It is necessary to strengthen the management of OTC drugs sold to adolescents and health education on self-medication for adolescents. The respondents included in this study were aged between 12 and 18 years, and this range included those in tertiary education and those who were not. Since both have changed dramatically in knowledge and lifestyle, the suggestions will be made towards health departments, drug manufacturers and distributors, media, schools and families for respondents with different education levels.

First, health authorities should establish a regulatory system, especially for adolescents who buy OTC drugs, pay attention to their self-medication needs, and make adolescents able to purchase the drugs they need with the help of pharmacists or other professional medical staff. Secondly, drug manufacturers should make the labels of OTC drugs more easily identifiable on the drug packaging and make it easier to explain how to use drugs scientifically and correctly in the outer packaging and instructions. Adolescents who do not receive higher education spend most of their time in the family and school environment. Therefore, for these adolescents, the role of schools and families in the scientific use of OTC drugs should be valued. Schools should pass more scientific knowledge of medication in class, and families should pass correct and scientific medication concepts. For those receiving higher education, health education on the scientific use of non-prescription drugs should be promoted in classrooms, associations, publicity posters and other forms on university campuses. In addition, these university students are exposed to many online media, so the media should pay attention to the accuracy of information when transmitting medication information. Health authorities should pay attention to and regulate online OTC drug purchase channels and strengthen the monitoring of online OTC drug sales. Finally, adolescents and their families should take the initiative to learn how to scientifically identify and use OTC drugs. In a randomized controlled trial in Japan, proactive use of self-medication knowledge provided by pharmacists was shown to be effective in promoting safe self-medication behavior among the public^50^; Other studies have shown that the drug use behavior of adolescents can be improved to a certain extent with the intervention of media^51^.

## Strengths and limitations of the study

### Strengths

This study has several strengths. Firstly, we utilized data from a cross-sectional survey conducted in the Chinese Mainland in 2021. Moreover, the survey employed a multi-stage sampling method to obtain a representative sample. Additionally, we combined family health, health literacy, and mental health status to analyze adolescents' non-prescription drug purchase behavior, which enriched the theoretical application value of health literacy in the field of adolescent self-medication. Targeted suggestions were provided for the future health promotion and health education practices of scientific medication in adolescents. Practical contributions were also made.

### Limitations

This study has several limitations. Firstly, the data is based solely on self-report questionnaires, which can be influenced by social expectations, self-report errors, and poor memory. Secondly, a cross-sectional design was used in this study. The results were only used to explore the factors associated with the dependent variable, which did not allow for causal inferences to be made based on the findings. Thirdly, the study participants were Chinese adolescents. As adolescence is a unique stage in life, the results of this study may not be generalizable to other countries. To address this limitation, future studies should aim to include participants from more diverse countries or regions. The behavior characteristics of adolescents and other critical factors they consider when purchasing OTC medications may change in subsequent years.

## Conclusion

Chinese adolescents aged 12–18 frequently use OTC drugs for self-medication. The likelihood of considering drug safety as essential was negatively correlated with monthly family income, while health literacy and family health status were positively correlated with considering drug efficacy and safety as essential. This study examines the variables related to the importance of considering the efficacy and safety of drugs during adolescent self-medication. The findings provide research ideas for future exploration of factors to be considered when standardizing follow-up studies of adolescent self-medication behavior. Additionally, the results can serve as a basis for relevant departments to formulate policies to regulate adolescent self-medication behavior. Further studies can investigate the variables associated with the significance of additional factors in adolescents' self-medication, as well as intervention models and methods to encourage rational self-medication in adolescents.

## Supplementary Information

Supplementary Tables.

## Data Availability

All data, models, and code generated or used during the study appear in the submitted article.
