# Publisher Correction: Status of self-medication and the relevant factors regarding drug efficacy and safety as important considerations among adolescents aged 12–18 in China: a cross-sectional study

**DOI:** 10.1038/s41598-024-73047-x

**Published:** 2024-09-26

**Authors:** Diyue Liu, Pu Ge, Xialei Li, Wenying Hong, Mengjie Huang, Lijun Zhu, Ayidana Kaierdebieke, Wenbian Yu, Jiale Qi, Keping Pu, Rong Ling, LuTong Pan, Xinying Sun, Yibo Wu, Qiqin Feng

**Affiliations:** 1 International School of Public Health and One Health, Hainan Medical University, Hiaikou, China; 2 School of Traditional Chinese Medicine, Beijing University of Chinese Medicine, Beijing, China; 3 School of Pharmaceutical Sciences, Shandong University, Jinan, China; 4 University of Macau, Macao, China; 5 School of Public Health, ShanDong University, Jinan, China; 6 College of Communication and Art Design, University of Shanghai for Science and Technology, Shanghai, China; 7 School of Public Health, Xi’an Jiaotong University, Xi’an, China; 8 Zhuhai Institute of Social Development, Zhuhai, China; 9 International School of Journalism and Communication, Zhengzhou University, Zhengzhou, China; 10 Institute of School of Nursing, China Medical University, Shenyang, China; 11 Jilin University School of Pharmaceutical Sciences, Jilin University, Changchun, China; 12 School of Public Health, Shandong University, Beijing, China; 13 School of Public Health, Peking University, Beijing, China

Correction to: *Scientific Reports *10.1038/s41598-024-59204-2, published online 01 May 2024

In the original version of this Article, Yibo Wu was omitted as a corresponding author. The correct corresponding authors for this Article are Yibo Wu and Qiqin Feng.

The original Article has been corrected.

