# Supplementary material for: Status of self-medication and the relevant factors regarding drug efficacy and safety as important considerations among adolescents aged 12–18 in China: a cross-sectional study
Source: Sci Rep. 2024 May 1;14:9982. doi: 10.1038/s41598-024-59204-2 (PMC11063147; doi:10.1038/s41598-024-59204-2)
Supplement: Supplementary file 1 — Supplementary Tables. [file 41598_2024_59204_MOESM1_ESM.docx]

**S1 Table.** **Variables and Assignment**

| **Type of**  **Variables** | | **Variables** | | **Assignment** |
| --- | --- | --- | --- | --- |
| Independent variables | | Location | | Eastern China =0， Middle China =1， Western China =2 |
|  | | Place of residence | | Rural =0， Urban=1 |
|  | | Monthly income | | ￥0-4500=0，≥￥4501=1 |
|  | | Gender | | Female=0，male=1 |
|  | | Ethnicity | | Han=0，Minorities=1 |
|  | | Education level | | Not in higher education =0， Undergoing higher education=1 |
|  | | Only child | | No=0，Yes=1 |
|  | | HLS-SF | | ≤33 =0(Low score group)，＞33 =1(High score group) |
|  | | FHS-SF^a^ | | ≤5=0(Low score group)，＞5=1(High score group) |
|  | | PHQ-9 | | ≤4=0(No depression)，＞4 =1(Depression) |
|  | | GAD-7 | | ≤4 =0(No anxiety)，＞4 =1(Anxiety) |
| Dependent variable | Have you ever purchased a kind of OTC drug | | | No=0， Yes =1 |
|  | | | Whether a drug's own properties are an important consideration in the purchase of an OTC drug^b^ | No=0， Yes =1 |

*a: The family health status of the participants was grouped according to the transformed scores of the Family Health Short Form*

*b:* *The attributes include drug price, drug efficacy, drug safety, drug taste, drug ease of use, drug packaging niceness, and drug dosage form. Each attribute was assigned a separate value for analysis purposes.*

**S2 Table. Univariate binary logistic regression of the self-attributes of OTC respondents had purchased**

| **Attribution** | **Variable** | | | | **β** | **SE** | ***P*** | **OR** | **95%CI** |
| --- | --- | --- | --- | --- | --- | --- | --- | --- | --- |
| **Drug price** |  | | | |  |  |  |  |  |
|  | **Location (control group = Eastern)** | | | |  |  |  |  |  |
|  | Middle | | | | 0.226 | 0.160 | 0.156 | 1.254 | 0.917-1.714 |
|  | Western | | | | **0.427** | **0.165** | **0.010** | **1.532** | **1.109-2.116** |
|  | **Place of residence (control group = Rural)** | | | |  |  |  |  |  |
|  | Urban | | | | **-0.358** | **0.146** | **0.014** | **0.699** | **0.525-0.930** |
|  | **Monthly income (RMB) a (control group =＜￥4500)** | | | |  |  |  |  |  |
|  | ≥￥4501 | | | | **-0.334** | **0.135** | **0.013** | **0.716** | **0.550-0.932** |
|  | **Gender (control group = Female)** | | | |  |  |  |  |  |
|  | Male  **Ethnicity (control group = Han)**  Minorities | | | | 0.210  0.121 | 0.135  0.245 | 0.120  0.620 | 1.233  1.129 | 0.947-1.606  0.699-1.824 |
|  | **Education level (control group = Not in higher education)** | | | |  |  |  |  |  |
|  | Undergoing higher education | | | | -0.049 | 0.141 | 0.728 | 0.952 | 0.722-1.255 |
|  | **Single-child (control group =No)** | | | |  |  |  |  |  |
|  | Yes  **Health literacy (control group = Low score group)**  High score group  **Family health (control group = Low score group)**  High score group | | | | **-0.423**  -0.277  -0.160 | **0.147**  0.143  0.148 | **0.004**  0.114  0.280 | **0.655**  0.797  0.852 | **0.491-0.874**  0.602-1.056  0.638-1.139 |
|  | **Depression (control group = No)** | | | |  |  |  |  |  |
|  | Yes | | | | **0.407** | **0.135** | **0.003** | **1.502** | **1.153-1.958** |
|  | **Anxiety (control group = No)** | | | |  |  |  |  |  |
|  | Yes | | | | **0.388** | **0.135** | **0.004** | **1.474** | **1.131-1.921** |
| **Drug efficacy** |  | | | |  |  |  |  |  |
|  | **Location (control group = Eastern)** | | | |  |  |  |  |  |
|  | Middle | | | | 0.097 | 0.156 | 0.535 | 1.102 | 0.811-1.497 |
|  | Western | | | | -0.057 | 0.162 | 0.726 | 0.945 | 0.688-1.298 |
|  | **Place of residence (control group = Rural)** | | | |  |  |  |  |  |
|  | Urban | | | | 0.259 | 0.144 | 0.071 | 1.296 | 0.978-1.718 |
|  | **Monthly income (RMB) a (control group =＜￥4500)** | | | |  |  |  |  |  |
|  | ≥￥4501 | | | | -0.115 | 0.131 | 0.379 | 0.891 | 0.690-1.152 |
|  | **Gender (control group = Female)** | | | |  |  |  |  |  |
|  | Male  **Ethnicity (control group = Han)**  Minorities | | | | 0.006  -0.326 | 0.132  0.239 | 0.962  0.173 | 1.006  0.722 | 0.776-1.304  0.452-1.154 |
|  | **Education level (control group = Not in higher education)** | | | |  |  |  |  |  |
|  | Undergoing higher education | | | | -0.152 | 0.138 | 0.269 | 0.859 | 0.656-1.125 |
|  | **Single-child (control group =No)** | | | |  |  |  |  |  |
|  | Yes  **Health literacy (control group = Low score group)**  High score group  **Family health (control group = Low score group)**  High score group | | | | 0.019  0.255  **0.471** | 0.140  0.141  **0.145** | 0.892  0.070  **0.001** | 1.019  1.291  **1.601** | 0.775-1.341  0.980-1.701  **1.206-2.127** |
|  | **Depression (control group = No)** | | | |  |  |  |  |  |
|  | Yes | | | | -0.228 | 0.131 | 0.082 | 0.796 | 0.615-1.030 |
|  | **Anxiety (control group = No)** | | | |  |  |  |  |  |
|  | Yes | | | | **-0.383** | **0.133** | **0.004** | **0.682** | **0.525-0.884** |
| **Drug safety** |  | | | |  |  |  |  |  |
|  | **Location (control group = Eastern)** | | | |  |  |  |  |  |
|  | Middle | | | | 0.205 | 0.162 | 0.205 | 1.228 | 0.894-1.686 |
|  | Western | | | | 0.259 | 0.171 | 0.130 | 1.295 | 0.927-1.810 |
|  | **Place of residence (control group = Rural)** | | | |  |  |  |  |  |
|  | Urban | | | | 0.268 | 0.148 | 0.070 | 1.307 | 0.978-1.748 |
|  | **Monthly income (RMB) a (control group =＜￥4500)** | | | |  |  |  |  |  |
|  | ≥￥4501 | | | | **-0.431** | **0.136** | **0.002** | **0.650** | **0.498-0.849** |
|  | **Gender (control group = Female)** | | | |  |  |  |  |  |
|  | Male  **Ethnicity (control group = Han)**  Minorities | | | | -0.138  -0.127 | 0.137  0.248 | 0.313  0.608 | 0.871  0.880 | 0.666-1.139  0.541-1.432 |
|  | **Education level (control group = Not in higher education)** | | | |  |  |  |  |  |
|  | Undergoing higher education | | | | 0.139 | 0.144 | 0.337 | 1.149 | 0.865-1.524 |
|  | **Single-child (control group =No)** | | | |  |  |  |  |  |
|  | Yes  **Health literacy (control group = Low score group)**  High score group  **Family health (control group = Low score group)**  High score group | | | | -0.273  0.190  **0.567** | 0.144  0.146  **0.148** | 0.057  0.191  **＜0.001** | 0.761  1.210  **1.763** | 0.575-1.008  0.909-1.609  **1.319-2.357** |
|  | **Depression (control group = No)** | | | |  |  |  |  |  |
|  | Yes | | | | 0.025 | 0.136 | 0.856 | 1.025 | 0.785-1.338 |
|  | **Anxiety (control group = No)** | | | |  |  |  |  |  |
|  | Yes | | | | -0.128 | 0.138 | 0.351 | 0.880 | 0.672-1.152 |
| **Drug taste** |  | | | |  |  |  |  |  |
|  | **Location (control group = Eastern)** | | | |  |  |  |  |  |
|  | Middle | | | | -0.420 | 0.255 | 0.099 | 0.657 | 0.399-1.082 |
|  | Western | | | | -0.414 | 0.267 | 0.121 | 0.661 | 0.391-1.116 |
|  | **Place of residence (control group = Rural)** | | | |  |  |  |  |  |
|  | Urban | | | | **-0.499** | **0.215** | **0.020** | **0.607** | **0.398-0.925** |
|  | **Monthly income (RMB) a (control group =＜￥4500)** | | | |  |  |  |  |  |
|  | ≥￥4501 | | | | **-0.447** | **0.214** | **0.037** | **0.640** | **0.420-0.974** |
|  | **Gender (control group = Female)** | | | |  |  |  |  |  |
|  | Male | | | | 0.188 | 0.208 | 0.364 | 1.207 | 0.804-1.814 |
|  | **Ethnicity (control group = Han)**  Minorities  **Education level (control group = Not in higher education)** | | | | -0.375 | 0.439 | 0.393 | 0.687 | 0.291-1.624 |
|  | Undergoing higher education | | | | **-1.064** | **0.274** | **<0.001** | **0.345** | **0.202-0.591** |
|  | **Single-child (control group =No)** | | | |  |  |  |  |  |
|  | Yes  **Health literacy (control group = Low score group)**  High score group  **Family health (control group = Low score group)**  High score group | | | | 0.020  **-0.826**  -0.254 | 0.221  **0.209**  0.222 | 0.928  **＜0.001**  0.253 | 1.020  **0.438**  0.776 | 0.662-1.573  **0.290-0.660**  0.502-1.199 |
|  | **Depression (control group = No)**  Yes | | | | -0.295 | 0.207 | 0.154 | 0.744 | 0.496-1.117 |
|  | **Anxiety (control group = No)** | | | |  |  |  |  |  |
|  | Yes | | | | -0.207 | 0.215 | 0.335 | 0.813 | 0.534-1.238 |
| **The convenience of drug use** | | |  | |  |  |  |  |  |
|  | **Location (control group = Eastern)** | | | |  |  |  |  |  |
|  | Middle | | | | -0.156 | 0.204 | 0.444 | 0.856 | 0.574-1.275 |
|  | Western | | | | -0.165 | 0.214 | 0.440 | 0.848 | 0.558-1.289 |
|  | **Place of residence (control group = Rural)** | | | |  |  |  |  |  |
|  | Urban | | | | 0.096 | 0.191 | 0.614 | 1.101 | 0.757-1.601 |
|  | **Monthly income (RMB) a (control group =＜￥4500)** | | | |  |  |  |  |  |
|  | ≥￥4501 | | | | 0.001 | 0.170 | 0.997 | 1.001 | 0.717-1.397 |
|  | **Gender (control group = Female)** | | | |  |  |  |  |  |
|  | Male  **Ethnicity (control group = Han)**  Minorities | | | | -0.056  -0.350 | 0.173  0.350 | 0.748  0.318 | 0.946  0.705 | 0.674-1.327  0.355-1.401 |
|  | **Education level (control group = Not in higher education)** | | | |  |  |  |  |  |
|  | Undergoing higher education | | | | 0.230 | 0.176 | 0.190 | 1.259 | 0.892-1.777 |
|  | **Single-child (control group =No)** | | | |  |  |  |  |  |
|  | Yes  **Health literacy (control group = Low score group)**  High score group  **Family health (control group = Low score group)**  High score group | | | | 0.139  -0.057  -0.090 | 0.179  0.183  0.187 | 0.439  0.757  0.631 | 1.149  0.945  0.914 | 0.808-1.632  0.660-1.352  0.633-1.319 |
|  | **Depression (control group = No)** | | | |  |  |  |  |  |
|  | Yes | | | | 0.293 | 0.173 | 0.090 | 1.340 | 0.955-1.880 |
|  | **Anxiety (control group = No)** | | | |  |  |  |  |  |
|  | Yes | | | | 0.320 | 0.171 | 0.061 | 1.377 | 0.985-1.924 |
| **Exquisite degree of drug packaging** | | | |  |  |  |  |  |  |
|  | **Location (control group = Eastern)** | | | |  |  |  |  |  |
|  | Middle | | | | -0.104 | 0.398 | 0.793 | 0.901 | 0.413-1.967 |
|  | Western | | | | 0.468 | 0.355 | 0.187 | 1.597 | 0.796-3.203 |
|  | **Place of residence (control group = Rural)** | | | |  |  |  |  |  |
|  | Urban | | | | -0.587 | 0.315 | 0.062 | 0.556 | 0.300-1.031 |
|  | **Monthly income (RMB) a (control group =＜￥4500)** | | | |  |  |  |  |  |
|  | ≥￥4501 | | | | -0.210 | 0.314 | 0.504 | 0.811 | 0.439-1.500 |
|  | **Gender (control group = Female)** | | | |  |  |  |  |  |
|  | Male  **Ethnicity (control group = Han)**  Minorities | | | | 0.165  -0.602 | 0.310  0.734 | 0.595  0.412 | 1.179  0.548 | 0.642-2.165  0.130-2.307 |
|  | **Education level (control group = Not in higher education)** | | | |  |  |  |  |  |
|  | Undergoing higher education | | | | -0.712 | 0.380 | 0.061 | 0.491 | 0.233-1.034 |
|  | **Single-child (control group =No)** | | | |  |  |  |  |  |
|  | yes  **Health literacy (control group = Low score group)**  High score group  **Family health (control group = Low score group)**  High score group  **Depression (control group = No)** | | | | -0.237  -0.477  **-1.306** | 0.346  0.315  **0.313** | 0.493  0.130  **＜0.001** | 0.789  0.621  **0.271** | 0.401-1.554  0.335-1.150  **0.147-0.500** |
|  | Yes | | | | 0.410 | 0.320 | 0.200 | 1.507 | 0.804-2.823 |
|  | **Anxiety (control group = No)** | | | |  |  |  |  |  |
|  | Yes | | | | 0.401 | 0.309 | 0.194 | 0.493 | 0.815-2.736 |
| **Drug dosage form** | |  | | |  |  |  |  |  |
|  | **Location (control group = Eastern)** | | | |  |  |  |  |  |
|  | Middle | | | | -0.180 | 0.189 | 0.341 | 0.835 | 0.576-1.210 |
|  | Western | | | | -0.004 | 0.191 | 0.982 | 0.996 | 0.684-1.449 |
|  | **Place of residence (control group = Rural)** | | | |  |  |  |  |  |
|  | Urban | | | | -0.004 | 0.173 | 0.984 | 0.996 | 0.711-1.398 |
|  | **Monthly income (RMB) a (control group =＜￥4500)** | | | |  |  |  |  |  |
|  | ≥￥4501 | | | | 0.032 | 0.156 | 0.837 | 1.033 | 0.760-1.402 |
|  | **Gender (control group = Female)** | | | |  |  |  |  |  |
|  | Male  **Ethnicity (control group = Han)**  Minorities | | | | -0.298  -0.534 | 0.161  0.336 | 0.064  0.112 | 0.742  0.586 | 0.541-1.017  0.304-1.132 |
|  | **Education level (control group = Not in higher education)** | | | |  |  |  |  |  |
|  | Undergoing higher education | | | | -0.171 | 0.168 | 0.307 | 0.843 | 0.607-1.170 |
|  | **Single-child (control group =No)** | | | |  |  |  |  |  |
|  | Yes  **Health literacy (control group = Low score group)**  High score group  **Family health (control group = Low score group)**  High score group | | | | 0.124  -0.266  -0.167 | 0.165  0.165  0.171 | 0.453  0.107  0.326 | 1.132  0.767  0.846 | 0.819-1.564  0.55-1.059  0.605-1.182 |
|  | **Depression (control group = No)** | | | |  |  |  |  |  |
|  | Yes | | | | 0.115 | 0.157 | 0.465 | 1.121 | 0.825-1.525 |
|  | **Anxiety (control group = No)** | | | |  |  |  |  |  |
|  | Yes | | | | 0.160 | 0.157 | 0.309 | 1.174 | 0.862-1.598 |

**S3 Table. Subgroup analysis: whether respondents consider drug efficacy as an important consideration in purchasing OTC drugs (gender)**

| **Subgroup** | **Variable** | **β** | **SE** | ***P*** | **OR** | **95%CI** |
| --- | --- | --- | --- | --- | --- | --- |
| **Gender** |  |  |  |  |  |  |
| Female | **Location (control group = Eastern)**  Middle  Western  **Place of residence (control group = Rural)**  Urban  **Monthly income (RMB) a (control group =≤￥4500)** | -0.383  0.055  **0.416** | 0.216  0.220  **0.205** | 0.076  0.803  **0.042** | 0.682  1.056  **1.516** | 0.446-1.041  0.686-1.627  **1.015-2.265** |
|  | ≥￥4501  **HLS-SF Healthcare (control group = Low score group)**  High score group | **-0.538**  **0.818** | **0.190**  **0.204** | **0.005**  **＜0.001** | **0.584**  **2.267** | **0.402-0.848**  **1.520-3.380** |
|  | **FHS-SF Family health resources (control group = Low score group)** |  |  |  |  |  |
|  | High score group | **0.461** | **0.185** | **0.013** | **1.586** | **1.103-2.279** |
| Male | **Location (control group = Eastern)** |  |  |  |  |  |
|  | Middle  Western  **Place of residence (control group = Rural)**  Urban | **0.536**  -0.339  0.077 | **0.250**  0.253  0.223 | **0.032**  0.181  0.730 | **1.709**  0.713  1.080 | **1.047-2.788**  0.434-1.171  0.698-1.671 |
|  | **Family healthy lifestyle (control group = Low score group)** |  |  |  |  |  |
|  | High score group | **0.465** | **0.216** | **0.032** | **1.591** | **1.041-2.431** |

**S4 Table. Subgroup analysis: whether respondents consider drug safety as an important consideration in purchasing OTC drugs (gender)**

| **Subgroup** | **Variable** | **β** | **SE** | ***P*** | **OR** | **95%CI** |
| --- | --- | --- | --- | --- | --- | --- |
| **Gender** |  |  |  |  |  |  |
| Female | **Location (control group = Eastern)**  Middle  Western  **Place of residence (control group = Rural)**  Urban  **Monthly income (RMB) a (control group =≤￥4500)** | -0.244  -0.117  0.173 | 0.227  0.229  0.214 | 0.283  0.609  0.421 | 0.783  0.889  1.188 | 0.502-1.223  0.567-1.394  0.781-1.808 |
|  | ≥￥4501 | **-0.625** | **0.199** | **0.002** | **0.535** | **0.363-0.790** |
|  | **FHS-SF Family health resources (control group = Low score group)** |  |  |  |  |  |
|  | High score group | **1.007** | **0.194** | **<0.001** | **2.737** | **1.872-4.001** |
| Male | **Location (control group = Eastern)**  Middle  Western  **Place of residence (control group =Rural)**  Urban  **Monthly income (RMB) a (control group =≤￥4500)** | **0.557**  **0.747**  **0.531** | **0.255**  **0.278**  **0.238** | **0.029**  **0.007**  **0.026** | **1.745**  **2.110**  **1.701** | **1.058-2.879**  **1.223-3.640**  **1.067-2.712** |
|  | ≥￥4501 | **-0.707** | **0.224** | **0.002** | **0.493** | **0.318-0.765** |
|  | **Family healthy lifestyle (control group = Low score group)** |  |  |  |  |  |
|  | High score group | **0.587** | **0.224** | **0.009** | **1.799** | **1.159-2.792** |

**S5 Table. Subgroup analysis: whether respondents consider drug efficacy as an important consideration in purchasing OTC drugs (location)**

| **Subgroup** | **Variable** | **β** | **SE** | ***P*** | **OR** | **95%CI** |
| --- | --- | --- | --- | --- | --- | --- |
| **Location** |  |  |  |  |  |  |
| Eastern | **Place of residence (control group =Rural)**  Urban  **Gender (control group = Female)**  Male  **HLS-SF Healthcare (control group =Low score group)** | 0.200  -0.027 | 0.212  0.195 | 0.346  0.891 | 1.221  0.974 | 0.806-1.851  0.664-1.427 |
|  | High score group | **0.505** | **0.213** | **0.018** | **1.657** | **1.092-2.514** |
|  | **FHS-SF Family health resources (control group = Low score group)** |  |  |  |  |  |
|  | High score group | **0.473** | **0.197** | **0.016** | **1.604** | **1.091-2.359** |
| Middle  Western | **Place of residence (control group = Rural)**  Urban  **Gender (control group = Female)**  Male  **Place of residence (control group =Rural)**  Urban  **Gender (control group =Female)** | 0.443  **0.659**  -0.116 | 0.293  **0.259**  0.298 | 0.131  **0.011**  0.698 | 1.558  **1.932**  0.891 | 0.877-2.768  **1.162-3.212**  0.496-1.598 |
|  | Male | **-0.552** | **0.273** | **0.043** | **0.576** | **0.337-0.984** |
|  | **Family healthy lifestyle (control group = Low score group)** |  |  |  |  |  |
|  | High score group | **0.731** | **0.288** | **0.011** | **2.078** | **1.182-3.652** |

**S6 Table. Subgroup analysis: whether respondents consider drug safety as an important consideration in purchasing OTC drugs (location)**

| **Subgroup** | **Variable** | **β** | **SE** | ***P*** | **OR** | **95%CI** |
| --- | --- | --- | --- | --- | --- | --- |
| **Location** |  |  |  |  |  |  |
| Eastern | **Place of residence (control group = Rural)**  Urban  **Gender (control group = Female)**  Male  **Monthly income (RMB) a (control group =≤￥4500)**  ≥￥4501  **Disease prevention (control group = Low score group)** | 0.392  **-0.428**  **-0.718** | 0.225  **0.203**  **0.218** | 0.081  **0.035**  **0.001** | 1.480  **0.652**  **0.488** | 0.952-2.301  **0.437-0.971**  **0.318-0.747** |
|  | High score group | **0.788** | **0.231** | **0.001** | **2.200** | **1.399-3.458** |
|  | **FHS-SF Family health resources (control group =Low score group)** |  |  |  |  |  |
|  | High score group | **0.689** | **0.213** | **0.001** | **1.992** | **1.312-3.025** |
| Middle  Western | **Place of residence (control group = Rural)**  Urban  **Gender (control group = Female)**  Male  **Monthly income (RMB) a (control group =≤￥4500)**  ≥￥4501  **FHS-SF Family health resources (control group = Low score group)**  high score group  **Place of residence (control group =Rural)**  Urban  **Gender (control group = Female)** | -0.407  0.364  **-1.097**  **1.091**  **0.733** | 0.352  0.289  **0.290**  **0.285**  **0.307** | 0.248  0.208  **＜0.001**  **＜0.001**  **0.017** | 0.666  1.439  **0.334**  **2.978**  **2.081** | 0.334-1.328  0.816-2.537  **0.189-0.590**  **1.702-5.211**  **1.140-3.799** |
|  | Male | 0.294 | 0.298 | 0.324 | 1.342 | 0.748-2.409 |
|  | **Family healthy lifestyle (control group = Low score group)** |  |  |  |  |  |
|  | High score group | **0.728** | **0.323** | **0.012** | **2.071** | **1.171-3.664** |

**S7 Table. Subgroup analysis: whether respondents consider drug efficacy as an important consideration in purchasing OTC drugs (place of residence)**

| **Subgroup** | **Variable** | **β** | **SE** | ***P*** | **OR** | **95%CI** |
| --- | --- | --- | --- | --- | --- | --- |
| **Place of residence** |  |  |  |  |  |  |
| Rural | **Location (control group = Eastern)**  Middle  Western  **Gender (control group = Female)**  Male  **HLS-SF Healthcare (control group = Low score group)**  High score group | -0.008  0.201  0.245  **0.681** | 0.306  0.304  0.248  **0.265** | 0.980  0.507  0.322  **0.010** | 0.992  1.223  1.279  **1.976** | 0.545-1.806  0.675-2.217  0.786-2.079  **1.175-3.322** |
| Urban | **Location (control group = Eastern)** |  |  |  |  |  |
|  | Middle  Western  **Gender (control group = Female)**  Male | 0.110  -0.170  -0.026 | 0.186  0.195  0.160 | 0.554  0.382  0.873 | 1.116  0.843  0.975 | 0.775-1.607  0.575-1.236  0.712-1.335 |
|  | **Family healthy lifestyle (control group = Low score group)** |  |  |  |  |  |
|  | High score group | **0.504** | **0.172** | **0.003** | **1.655** | **1.182-2.317** |

**S8 Table. Subgroup analysis: whether respondents consider drug safety as an important consideration in purchasing OTC drugs (place of residence)**

| **Subgroup** | **Variable** | **β** | **SE** | ***P*** | **OR** | **95%CI** |
| --- | --- | --- | --- | --- | --- | --- |
| **Place of residence** |  |  |  |  |  |  |
| Urban | **Location (control group = Eastern)**  Middle  Western  **Gender (control group = Female)**  Male  **Monthly income (RMB) a (control group =≤￥4500)**  ≥￥4501  **FHS-SF Family health resources (control group = Low score group)**  High score group | -0.149  0.381  0.023  **-0.787**  **0.882** | 0.196  0.217  0.172  **0.175**  **0.175** | 0.446  0.080  0.894  **＜0.001**  **＜0.001** | 0.861  1.463  1.023  **0.455**  **2.416** | 0.587-1.264  0.956-2.241  0.731-1.432  **0.323-0.642**  **1.716-3.401** |
| Rural | **Location (control group = Eastern)** |  |  |  |  |  |
|  | Middle  Western  **Gender (control group = Female)**  Male  **Single-child (control group =No)**  Yes | 0.980  0.056  -0.223  **-0.830** | 0.347  0.304  0.262  **0.379** | 0.005  0.855  0.395  **0.028** | 2.665  1.057  0.800  **0.436** | 1.349-5.262  0.582-1.920  0.479-1.337  **0.207-0.916** |
|  | **Health promotion(control group = Low score group)** |  |  |  |  |  |
|  | High score group | **0.733** | **0.306** | **0.017** | **2.081** | **1.142-3.790** |
